# Water fluoridation today: – benefits and challenges

**DOI:** 10.3389/froh.2026.1745916

**Published:** 2026-03-25

**Authors:** Andrew Rugg-Gunn, Ray Lowry, Barry Cockcroft, Anthony Damien Walmsley

**Affiliations:** 1 Professor Emeritus, Newcastle University, Newcastle upon Tyne, United Kingdom; 2 Retired, Department of Child Dental Health, Newcastle University, Newcastle upon Tyne, United Kingdom; 3 Retired, Consultant in Public Health, Newcastle Health Authority, Newcastle upon Tyne, United Kingdom; 4 Retired, Chief Dental Officer, Department of Health, London, United Kingdom; 5 Professor Emeritus, School of Dentistry, College of Medicine and Health, The University of Birmingham, Birmingham, United Kingdom

**Keywords:** dental caries, dental fluorosis, economic evaluation, fluoridation and public opinion, fluoridation and the environment, fluoride and health, health equity, health inequalities

## Abstract

The aim of this review is to describe the role of water fluoridation in preventing dental caries worldwide. Community water fluoridation began in 1945 and over the past 20 years has reached over 400 million people. In several countries, such as USA, Australia, New Zealand, Ireland, Singapore, Hong Kong, Brunei, Malaysia, Oman, Chile, Gabon and Brazil, a high proportion of the population receives fluoridated water. The clinical effectiveness is well established. Both water fluoridation and fluoride-containing toothpaste are effective and each adds to the effect of the other. Coverage of water fluoridation is equitable so that those at greatest risk of dental caries benefit most. Water fluoridation is cost-effective and has low environmental impact. Below the usual permitted concentration of 1.5 mgF/L, there is no risk to health. Prevalence of dental fluorosis is higher in fluoridated communities but severity is mild to very mild and of little aesthetic concern. In this regard, advice should focus on early use of fluoride-containing toothpaste to reduce fluoride ingestion during infancy and early childhood. The cost of water fluoridation is low. With increasing global urbanisation and improved equipment, the future of water fluoridation is promising. The WHO urges member states to consider implementing community water fluoridation.

## 1 Introduction

The prevalence and severity of dental caries has changed much during the past 80 years (1). The rise in the availability and affordability of sugar and high-sugar products, aided by aggressive marketing, led to catastrophic levels of caries in many countries (1, 2). But then the remarkable ability of fluoride to diminish caries severity was discovered and applied, first in public water supplies and then in toothpastes, mouthrinses and other vehicles (3). With its expanding use, fluoride, in its many forms aided by constant research, has improved oral health enormously in most parts of the world (1). Fluoride is a great success story but the story is not yet complete. The Director-General of WHO pointed out that oral diseases are among the most common noncommunicable diseases worldwide and that this burden is increasing particularly in low- and middle-income countries (4). About 3.5 billion people worldwide were affected by oral diseases in 2019, making them the most widespread conditions among more than 300 diseases and conditions that affect humanity (5, 6). In 2017, over 530 million children had untreated caries in primary teeth and over 2.3 billion people untreated caries of permanent teeth (7). Untreated dental caries was the most prevalent disease in WHO African Region in 2019 (8) with 29% of the population >5 years suffering from untreated caries of their permanent teeth. In primary teeth, untreated caries was the single most common chronic childhood disease in AFRO, with 39% of children aged 1–9 years in the Region estimated to be affected by untreated caries in 2019.

The impact of oral disease, of which dental caries is the most significant, is of social and economic importance. Pain and discomfort associated with oral diseases make concentration difficult, can cause people to miss school or work, and can lead to social isolation (4). The global economic burden of dental diseases was quantified by Righolt et al. (9) in 2015 as 544 billion USD – 357 billion USD as direct cost and 187 billion USD as indirect costs. Oral diseases remain a major public health issue for high-income countries, where expenditure on treatment often exceeds that for other diseases, including cancer, heart disease, stroke, and dementia. This is disturbing, given that much of the oral disease burden in high-income countries is due to dental caries and its complications, and this is preventable through the use of fluoride and other cost-effective measures (10). In England, in 2017–18, there were 45,077 children and adolescents admitted to hospital for extraction of decayed teeth, making dental decay by far the most common reason for children aged between five and nine to be admitted to hospital (11).

During the past 50 years it has been established that dietary sugar is the cause of dental caries, and of many other NCDs (1, 2). Governments now have policies to reduce sugar consumption (4). Removal of free sugars from our diet and to maximise appropriate use of fluoride are complementary strategies and essential if dental caries is to be controlled. This article aims to show that the adjustment of fluoride concentration in drinking water to the optimum has benefitted, is benefitting and will benefit oral health worldwide. With water fluoridation schemes active in 25 countries and reaching over 400 million people in 2004 (12), it is one of the main ways of providing caries protection. The WHO Global Oral Health Status report pointed out that there is a body of scientific evidence on the safety, efficacy, cost-effectiveness and population-wide feasibility of different fluoride vehicles. Fluoride toothpaste and water fluoridation are among the population-level fluoridation interventions with the strongest evidence (6). There are infrastructure requirements for water fluoridation and public health requirements to monitor effectiveness and safety. Regular use of well-formulated fluoride-containing toothpastes is encouraged by WHO (4). Water fluoridation and fluoride toothpaste are not alternatives; both provide benefit and their effects are additive: “additive” meaning that fluoride-containing toothpastes have a caries-preventive effect in fluoridated communities (13) and water fluoridation has a caries-preventing effect in populations where fluoride-containing toothpastes are widely available (14). The cost-benefit is substantial (6).

The history of water fluoridation is well recorded, beginning in the USA in 1945 (15). In several countries, water fluoridation is almost universal, but this depends on availability of a public drinking water system and technical expertise. Both restraints are changing: urbanisation is spreading across the globe and, with that, provision of public water systems and electricity. Technical skills to provide public utilities improve globally, aiding ability to operate water fluoridation systems. Equipment to adjust fluoride levels in water supplies improve, with some countries installing fluoridation equipment in small communities, not considered economic in years past.

While most water supplies contain fluoride at too low a concentration to provide benefit, a sizable population in the world drink water containing fluoride at too high a concentration, harmful to health. WHO gives guidance on this and on how to determine the concentration which will benefit oral health without adversity (16). This review will consider current effectiveness of water fluoridation at preventing dental caries, implementation costs, water fluoridation and health, and political and administrative aspects.

## 2 Extent of water fluoridation

Table 1 shows countries with community water fluoridation (CWF) programmes. There were no programmes in the 16 low-income countries (World Bank classification). The “population served” figures do not include sizable populations living in communities drinking water naturally containing optimum (or higher) concentrations of fluoride. Hong Kong and Singapore have complete coverage of their populations. In 14 countries more than half the population is supplied with fluoridated water. The global coverage is estimated to be 467 million or 6% of global population. The table is based on the British Fluoridation Society *One in a million* (12), other publications (152–155) and personal communications. It was difficult to contact countries when compiling this table and its contents should be taken as the best information available.

**Table 1:** Community water fluoridation programmes in high- and middle-income countries classed according to world bank (17) and as used by WHO.

High income	Pop. served	% of Pop. served	Middle income	Pop. served	% of Pop. served
Australia	24 mil.	89	Brazil	156 mil.	73
Brunei	457,000	98	Fiji	342,000	37
Canada	15.9 mil.	40	Gabon	2.1 mil.	80
Chile	11.0 mil.	55	Kiribati	59,000	43
Guyana	245,000	29	Malaysia	25.3 mil.	70
Hong Kong	7.5 mil.	100	Papua New Guinea	600,000	6
Ireland	3.4 mil.	64	Philippines	1.06 mil.	1
Korea	3.1 mil.	6	Vietnam	3.5 mil.	3
New Zealand	2.9 mil.	55			
Oman	3.1 mil.	56			
Panama	671,000	15			
Puerto Rico	35,000	1			
Singapore	6.0 mil.	100			
Spain	4.3 mil.	9			
United Kingdom	5.8 mil.	8			
USA	190 mil.	55			
*278.4 million*			*189 million*		

## 3 Effectiveness of water fluoridation

### 3.1 Measurement of clinical effectiveness

The occurrence of caries in populations can be expressed in several ways. Prevalence is the proportion of population affected, so that water fluoridation may decrease this proportion. Caries severity is measured by the dmf/DMF index, usually expressed as the mean number of teeth with caries experience per subject. Information on both prevalence and severity are useful although, in most reviews, clinical effectiveness has been expressed as percent caries reduction (PCR) or prevented fraction (PF) in caries severity – e.g., a PCR or PF of 30% in DMFT. This statistic is widely used in other oral health community programmes and will be given emphasis here.

The design of studies to investigate the clinical effect of water fluoridation has varied. Early studies were “before-and-after” studies, also known as historical control studies – how much caries experience fell after a water fluoridation scheme was introduced. This design is weak as background changes in caries experience, without water fluoridation, are usually unknown. A better design is the “parallel control” design, where a control community is included which is not exposed to water fluoridation. Caries experience is recorded in both fluoridated and non-fluoridated communities before fluoridation begins as well as a specified time, e.g., 5 years, after fluoridation. To allow valid conclusions, caries experience should be very similar in the two communities before fluoridation (baseline). This design is favoured by the Cochrane reviews of water fluoridation (14, 18) and exemplified in the recent UK CATFISH study (19). This design is very suitable for evaluating effectiveness of new fluoridation programmes on caries experience in children, but becomes untenable when follow-up periods are long, e.g., to evaluate effectiveness in adults, since (a) people move their residence, (b) maintaining contact with people who remain in the area over long time periods is very difficult, and (c) the non-fluoridated community may decide to fluoridate. It is also unsuitable for monitoring continuing clinical effectiveness in on-going programmes, which now may be the most relevant need.

There has been recent progress in evaluation of on-going water fluoridation programmes due to advances in statistical methods and computing power. In the past, cross-sectional and longitudinal observational studies could indicate relationships but not causation. This has now changed with development of “causal inference” from observational data (20, 21). Causal inference analysis of existing data was developed in economics, with the 2021 Nobel Prize awarded for “what conclusions can be drawn from natural experiments. Their approach has spread to other fields and revolutionised empirical research” (21). Indeed, this approach is increasingly used in public health research (22, 23). A review of the use of causal inference analysis in oral health research indicated that its use has been limited and it should be used more widely (24). “Time to move forward” has been promoted by Listl et al. (21) and Schuch et al. (25). Those evaluating community water fluoridation should accept that there are now valid methods other than those used by Cochrane (14, 18) – a pluralistic approach is required (26, 27).

### 3.2 Reviews of clinical effectiveness

Clinical effectiveness has been measured since the beginning of community water fluoridation 80 years ago. All known evaluations were summarised by Rugg-Gunn and Do (28). Prior to 1990, 113 studies (66 for primary and 86 for permanent teeth) showed high levels of effectiveness, while post-1990 and up until 2010, the 59 studies (30 in primary and 53 in permanent teeth) showed effectiveness but less than previously. This declining size of effect was reported in the two Cochrane reviews (14, 18). For example, the 2024 Cochrane Review stated: “Contemporary studies indicate that initiation of CWF may lead to a slightly greater reduction in dmf and may lead to a slightly greater increase in the proportion of caries-free children, but with smaller effect sizes than pre-1975 studies.” This was attributed to decreasing caries severity in these populations, principally due to the widespread introduction of fluoride-containing toothpastes in the 1970s. Nevertheless, CWF is still effective in children (29–33) and adults (33–39) and has been endorsed by authorities throughout the world (40–47). A recent article, written on behalf of FDI World Dental Federation endorsed CWF, discussed misconceptions and pointed out that CWF was a preventive tool not a cure-all (48). In the past, the USA has strongly advocated CWF (49) but in 2025, the US CDC was ordered to cease endorsement, under considerable pressure from the US Health and Human Services (HHS) Secretary, Robert F Kennedy Jr (50).

While caries occurrence has been by far the most widely recorded outcome, the sequelae are of medical, social and economic importance. In the UK, for many years tooth decay has been the main reason for hospital admission for children between 5 and 9 years old. In 2019, apart from the trauma of tooth extraction and hospitalisation, the estimated costs for hospital tooth removal due to tooth decay for children aged between 0 and 19 was 33 million GBP (51). However, Public Health England (51) reported that hospital admissions for decayed tooth extraction in children under 19 was 60% lower in areas with the highest concentration of fluoride in drinking water, compared with areas with the lowest levels of fluoride. The greatest reduction associated with water fluoridation in hospital admissions was seen in the most deprived areas. Similar findings were reported in Australia (52). Recording of sequelae is not new, as can be seen in Table 2 where prevalence of dental abscesses, and experience of toothache, tooth extraction and general anaesthetic for dental extraction, as well as costs were recorded (53). While DMFT/dmft has been the traditional way of summarising dental caries experience and clinical effectiveness, there have been calls for outcome measures to be policy-relevant (54) with recommendations to use generic health outcomes such as disability-adjusted life years and quality-adjusted life years.

**Table 2:** The effect of water fluoridation in 5-year-old children in the NE of England in 1976 on the occurrence of dental abscesses, toothache, general anaesthetics for tooth extraction, and cost of dental treatment (GBP, 1976) (53).

Sequelae of dental caries	Fluoridated area	Non-fluoridated area
% with 1 or more abscesses	0	5
% ever had toothache	17	38
% ever had GA for dental extraction	7	22
Cost of treatment already completed	£ 1.27	£ 1.63
Cost of treatment still required	£ 1.93	£ 7.89

### 3.3 Equity

**Figure 1:**
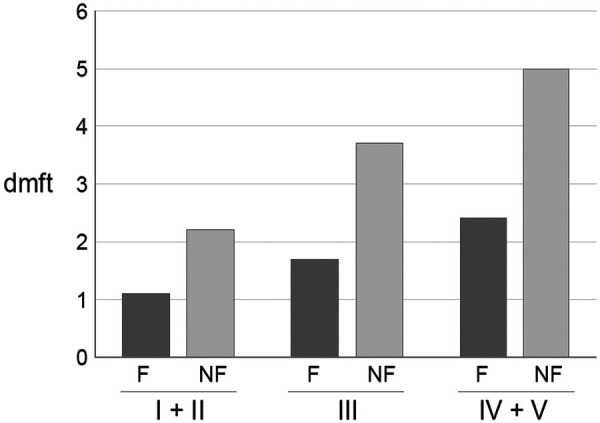
Caries experience (mean dmft) of 5-year-old children in fluoridated (F) and non-fluoridated (NF) areas in NE England in 1987 in three social class groupings (Registrar General's classification 1987) (55). I + II high social class; III middle social class; IV + V low social class.

A unique advantage of community water fluoridation is that it benefits people who are difficult to reach with other oral health preventive programmes, who are usually the people with the greatest need. Exposure to fluoride incorporated into aspects of everyday living, such as public drinking water, requires no behavioural change and has the potential to reduce inequalities as it is available to everyone, regardless of age, oral health behaviour, access to dental care, or socio-economic circumstances. In most populations, disease, including dental caries, is more prevalent and severe in socially deprived children and adults. These groups benefit most from water fluoridation (Figure 1) (55). In this table, while the prevented fractions are roughly similar for the three social class groupings (49%, 54%, 51% for social groups I + II, III, IV + V, respectively), the difference in the mean number caries-affected teeth per child between fluoridated and non-fluoridated communities was over twice as great (2.6 dmft compared with 1.1 dmft) in the most deprived children than in the least deprived children (55). On the other hand, the 2024 Cochrane Review stated that only one study, conducted after 1975, reported disparities according to socioeconomic status, with no evidence that deprivation influenced the relationship between water exposure and caries status (14). To what extent, different methods of recording deprivation explain these differences in conclusions is unclear. In the study of Carmichael et al. (55) the socioeconomic status of the individual was recorded, while most other studies used indices of area deprivation, which might dilute social disparities. A recent national Australian study reported substantially greater benefit for low social groups than for higher social groups where deprivation (income and education) had been recorded at the individual person level (56).

### 3.4 Cost-benefit

The high prevalence worldwide of dental caries imposes a significant economic burden both to the individual and society (57–59). Water fluoridation has the potential to reduce this burden and virtually all studies record the economic advantage of water fluoridation (57, 58). Although the 2024 Cochrane Review (14) did not include economic evaluation, it included a brief commentary based on 25 eligible reports. The Review stated that CWF appeared to offer good value for money due to its low per capita intervention delivery costs, potential to reduce caries, even at low magnitudes of effect size, and the related impact on dental treatment costs averted. The magnitude of cost-effectiveness (or net cost-savings) was shown to be sensitive to the size of the fluoridated population, the magnitude of water fluoride's effectiveness observed in more recent studies, and the underlying caries risk in the treated population. In the UK CATFISH study (19) where caries experience was low and caries reductions associated with water fluoridation were small, the cost-benefit was still in favour of water fluoridation (60). In the USA, Ran and Chattopadhyay (58) undertook an economic evaluation of community water fluoridation in the USA, where about two-thirds of households receive fluoridated water, on behalf of the national Center for Disease Control (CDC), Atlanta. They concluded that recent evidence continued to indicate that the economic benefit of community water fluoridation exceeded the intervention cost, and that the benefit-cost ratio increased with the community population size (58). In Ireland, with extensive water fluoridation, a recent economic evaluation concluded that CWF remained a cost-effective public health intervention for Irish schoolchildren (61).

The above reports explain that the magnitude of cost-benefit reduces with decreasing size of community supplied. This topic has been examined in Australia and New Zealand where equity is an important public health commitment (62). In New Zealand, Wright et al. concluded that fluoridation was cost-saving (dental costs exceeded fluoridation costs) for communities above about a thousand people (63). In Australia, Cobiac and Vos (64) comment that isolated communities may be disadvantaged groups with higher caries experience and that there may be good justification to extend coverage to include communities with population of <1,000 people, given the substantial dental disparities and inequalities in access to dental care that currently exist.

## 4 Water fluoridation and health

Community water fluoridation is likely to be the most investigated public health measure. While the beneficial effect of CWF on health should not be underestimated, the possibility of adverse health effects must be assessed and considered. Like most elements or compounds, fluoride is a poison if ingested in large amounts. Fluoride occurs naturally in community water supplies and the amount of fluoride added to bring concentration to the optimum is very small – the target is usually to bring the concentration to 0.5 to 1.0 mg fluoride per litre of water (mgF/L or ppm).

Over the past 80 years of CWF, there have been many investigations into possible adverse health effects; these have usually been undertaken by government-funded committees who have the expertise to instigate investigations and assess scientific data. Universally, these conclude that at the optimum concentration (0.5 to 1.0 mgF/L) there are no adverse health effects. Many of these national committees, though, set an upper limit on fluoride concentration in drinking water, usually 1.5mgF/L. If drinking water contains fluoride at a concentration higher than this upper limit, commonly, there is an obligation for the water supplier to reduce the concentration.

Because fluoride has an affinity for bone and the thyroid, these have been the most thoroughly investigated, and no adverse effects observed. Over the past 10 years, however, emphasis has shifted to possible effect on the growing brain, particularly focusing on IQ (Intelligence Quotient). This debate is ongoing. The most significant publication has been Green et al. (65). They concluded that “In this prospective birth cohort study from 6 cities in Canada, higher levels of fluoride exposure during pregnancy were associated with lower IQ scores in children at age 3 to 4 years. These findings were observed at fluoride levels typically found in white North American women. This indicated the possible need to reduce fluoride intake during pregnancy.” This investigation followed several reports on fluoride neurotoxicity in China and India, in districts with high levels of exposure to fluoride. These early studies were reviewed by Choi et al. (66): many of these 27 studies were poorly controlled and with fluoride intakes with little relevance to community water fluoridation.

The above study by Green et al. (65) has been heavily criticised (67). First, the study they used to supply their data (Canadian Maternal-Infant Research on Environment Chemicals, MIREC) focused on the prenatal exposure to “priority environmental chemicals”, and this list did not include fluoride. It was subsequently that Green et al. analysed spot urine samples collected during pregnancy for fluoride. The validity of using urinary fluoride excretion as a marker of fluoride ingestion has been studied (68) with the conclusion that urinary fluoride is a suitable marker of fluoride intake for groups but not for individuals (69, 70). These conclusions applied to 24 h urinary collections, not spot samples as used by Green et al. (65). Spot samples are a one-time void and, as Aylward et al. (71) pointed out, are not a reliable guide to 24 h urine excretion: “Because of substantial within- and between-individual variation in urinary flow and creatinine excretion rates, as well as the rapid urinary elimination pharmacokinetics of fluoride, concentrations of fluoride in individual spot samples may vary substantially even when underlying exposure rates are consistent and within the exposure guidance values.” (71). The use of the Wechsler scale of intelligence by Green et al. (65) to assess IQ has also been criticised (67, 72).

The above studies have adversely influenced decisions on CWF in the USA, resulting in the States of Florida and Utah deciding to cease their water fluoridation programmes (50). The US Environmental Protection Agency (73) and in 2021 the US National Academies of Sciences, Engineering and Medicine Committee (74) examined these issues, and the latter removed the classification of fluoride as a “presumed neurodevelopmental hazard” from its assessment.

An influential review was published by Taylor et al. (75): a systematic review and meta-analysis of epidemiological studies investigating children's IQ scores and prenatal or postnatal fluoride exposure. They found inverse associations and a dose-response association between fluoride measurements in urine and drinking water and children's IQ across the large multi-country epidemiological literature, adding that there were limited data and uncertainty in the dose-response association between fluoride exposure and children's IQ when fluoride exposure was estimated by drinking water alone at concentrations less than 1.5 mg/L.

This publication and information examined by the above US committees and other recent publications have been reviewed by Kumar et al. (76). They assessed the suitability of available cohort studies for modelling fluoride concentration in urine and water and IQ response and concluded that the data quality assessment revealed serious flaws that rendered the maternal urinary studies unacceptable for hazard assessment and benchmark dose modelling. The maternal urinary fluoride datasets did not show a homogenous response, and the neurodevelopmental hazard had not been adequately demonstrated. Kumar et al. (76) also commented that recent studies from Sweden, China, Canada, Denmark and Australia had not shown deficits in cognitive scores at low levels of fluoride exposure, and that IQ scores did not improve after the cessation of water fluoridation in Calgary, whereas there was a detrimental effect on dental caries outcomes (77, 78). While most studies included in Kumar's analysis (76) compared fluoridated with non-fluoridated communities, data from Denmark compared communities with naturally fluoridated water (at 0.81mgF/L) with fluoride-low water.

As recommended by Kumar et al. (76), an appropriate way to examine the relation between CWF and neurodevelopment is to examine IQ in communities with and without CWF at the level optimum for that community (between 0.5 to 1.0 mgF/L). Five such studies have been published (65, 72, 79–81). Two of these studies showed no difference in IQ between fluoridated and non-fluoridated communities, and the other three showed slightly higher IQ in the fluoridated communities (82).

Despite this reassurance from these epidemiological studies, often nationwide, of CWF at the optimum level in that community, there is pressure to lower the legal limit of 1.5 mgF/L due to neurodevelopmental risk during pregnancy (83). The European Commission asked EFSA to update its previous consumer risk assessment of fluoride after recent studies suggested a possible link to harmful effects on the developing nervous system of children. EFSA's experts also looked at the potential effects of fluoride on the thyroid, bones, and teeth. This report concluded that the 1.5mgF/L limit was sufficient for these entities. Over 20,000 scientific papers of relevant human and animal studies up to 2024 were reviewed. The report concluded that with current concentrations of fluoride in European drinking water, total fluoride exposure did not exceed the new safe and tolerable upper intake levels for almost all age groups and therefore did not pose a health concern; the one exception being children aged 4–8 years. For them, assuming typical fluoride concentrations in drinking water and a very conservative estimate of 100% ingestion of dental care products, mild dental fluorosis may occur in molar teeth, which are in development during this age. This is unlikely to occur if children spit the toothpaste out properly after brushing their teeth (83).

EFSA's experts set a safe level of intake of 3.3 mg/day for pregnant women and all age groups over 8 years of age, derived from potential effects on the developing central nervous system of the foetus. These intakes occur at drinking water levels over 1.5 milligrams per litre (mg/L). The report concluded that at levels of fluoride below 1.5 mg/L, the evidence for potential links were inconsistent and insufficient to draw clear conclusions, and that this safe level of intake was also protective of other potential adverse effects on bones and the thyroid (83).

In conclusion, health is not at risk when water fluoride levels are below the permitted level of 1.5 mg/L. These assessments include bone, thyroid and neurodevelopment. As fluoride levels increase above that threshold, health risks become more likely. Many of the studies reporting adverse effects on neurodevelopment were low quality and their validity has been questioned. These conclusions are made considering total fluoride exposure, fluoride in water, foods, and toothpaste; in early life, the main source of ingested fluoride is toothpaste (83).

## 5 Water fluoridation and dental fluorosis

Dental fluorosis is hypoplasia or hypomaturation of tooth enamel produced by chronic ingestion of excessive levels of fluoride during the period when teeth are developing. Although both primary and permanent teeth can be affected, prevalence is higher in permanent teeth for any given water fluoride level. In the permanent dentition, maxillary teeth are more affected than mandibular teeth, with prevalence highest in maxillary central incisors (84). Because of the aesthetic importance of permanent maxillary incisor teeth, the ages at which they are susceptible to develop fluorosis have been studied (84, 85). Although the first two years of life were generally found to be more important compared with later years, fluoride intake during each individual year (until the fourth year of life) was associated with fluorosis in these teeth. Subjects with the highest levels of fluoride intake during the whole of the first three years of life had the highest levels of fluorosis. Thus, the development of dental fluorosis appears to be related not only to the timing of intake but also to the cumulative duration of fluoride exposure (85–87).

Over a century ago, reports of “opaque, discoloured and disfigured” teeth were linked to geographical areas (88) and then water supply (89). The discovery that fluoride in water supplies was the cause (90) was followed by intense research led by Dean (91, 92) in many US States with varying concentrations of fluoride naturally in drinking water. In preparation for these extensive surveys, Dean (93) developed an index for assessing prevalence and severity of mottled enamel. Although modified (94), this index is still widely used today and recommended by WHO (95). It was recognised early (96, 97) that ambient temperature influenced the prevalence of dental fluorosis for any given level of fluoride in drinking water (more water being drunk in hotter climates) and, later, nutritional status (98, 99), with poor nutritional status associated with higher prevalence of dental fluorosis.

It was also recognised early on that there are causes of dental opacities and hypoplasia other than intake of fluoride (98), leading to development of two types of index – those which attempted to only record defects considered to be caused by fluoride, such as Dean's, and those which recorded all defects on the grounds that it was not possible to allocate cause (Table 3). This latter point was made by Forrest (106) who said: “severe degrees of fluoride mottling were unmistakable but in milder cases the cause of enamel defects cannot always be stated with certainty”. To give clarity to Dean's index, Horowitz et al. (104) and Horowitz (105) suggested modifications with their TSIF (Tooth Surface Index of Fluorosis). The first index to record all defects was by Al-Alousi et al. (100) to be followed by the DDE (Defects of Dental Enamel) index proposed by FDI (102). This was simplified by Clarkson and O'Mullane (103) and has been much used, although cumbersome to report. Thylstrup and Fejerskov (101) disagreed with this approach and proposed a fluorosis index where visual appearance was related to histological state. This index has been widely used due to its emphasis on milder grades of fluorosis. While a plethora of indices have been proposed and used, diagnosis and visual impact are much affected by whether teeth are examined wet (with saliva) or dry, and the degree of drying, and whether examined in natural light or artificial light of various intensities, or from photographs.

**Table 3:** Recording of dental enamel opacities and defects divided into those which record all defects and those which record dental fluorosis.

All defects	Dental fluorosis only
Al-Alousi (100)	Dean's (94)
Murray and Shaw (84)	Thylstrup and Fejerskov (TFI) (101)
Developmental Defects of Enamel (DDE) (102, 103)	Tooth Surface Index of Fluorosis (TSIF) (104, 105)

The above information indicates the difficulties in identifying and recording dental fluorosis (defects caused by excessive fluoride); it is a further step to know what level of prevalence and severity is of concern to the person examined. Are they upset by the aesthetic appearance of their teeth, usually their permanent maxillary anterior teeth? Acceptance or concern have been assessed in many ways since the early ‘90s (107, 108) – by parents and children assessing the child's teeth, visually or from photographs, using grades of satisfaction with appearance, or quality of life indices. Chankanka et al. (109) reviewed 35 reports. They concluded: (a) viewing photographs exaggerated the severity of dental fluorosis, (b) studies where parents and children viewed the child's teeth showed that mild or very mild dental fluorosis were rated similar to no fluorosis, (c) very mild and mild fluorosis had little effect of quality of life measurements with one study showing mild fluorosis preferable to no fluorosis, (d) mild fluorosis was sometimes associated with improved oral health-related quality of life, probably due to the public's greater emphasis on white teeth (109).

Most assessments of dental fluorosis in permanent maxillary incisor teeth have been made at age 8 to 10 years when these teeth are newly erupted. However, as the histological images of Thylstrup and Fejerskov (101) showed, the mildest forms of dental fluorosis are superficial. Abrasion and erosion are likely to reduce prevalence and severity, as shown by Do et al. (110). In their study, dental fluorosis was assessed with the TF index in a national oral health survey in 2003–4 when the children were aged 8–13 years. 314 of the same children were re-examined 8 years later using the same index. Over 60% of teeth scored TF1 at baseline were scored TF0 eight years later, and 66% of teeth scored TF2 or 3 at baseline were scored TF0 or 1 at follow-up – very mild and mild dental fluorosis can diminish over time. A similar observation was recorded first by Wong et al. (111) in Hong Kong children observing a decline in diffuse opacities, and then by Curtis et al. (112) and Levy (113) in their follow-up of children participating the Iowa birth cohort study on fluoride intake. They observed a statistically significant decline in generalized fluorosis severity scores and overall prevalence at later ages across all three measures of generalized fluorosis severity and commented that this trend should be accounted for when estimating the prevalence of fluorosis in a population using fluorosis severity data collected in children and adolescents (113).

Dean and others chose 1 ppm fluoride (1mgF/L) as the optimum concentration in drinking water in a temperate climate as it was associated with negligible levels of dental fluorosis (10% having mild or very mild fluorosis). However, during the 1980s, reports appeared that the prevalence and severity of dental fluorosis was increasing (114–116): this was in the USA with extensive water fluoridation and widespread use of fluoridated toothpaste. There followed many studies on sources of fluoride intake in children, and refining of optimal levels of fluoride intake (85). Both fluoridated water and fluoride toothpaste were major risk factors, with fluoride supplements and infant formula feeds important in the USA. The role of fluoride toothpaste became clear as the prevalence and severity had increased in both fluoridated and non-fluoridated communities. The age that toothbrushing with a fluoride toothpaste began was a dominant risk factor with some studies suggesting beginning before 12 months and others 24 months as significant. Risk increased with increasing concentration of fluoride in the toothpaste, with frequency of brushing and amount of toothpaste used as significant but less important factors (85, 117).

There followed much debate on (a) whether toothpastes containing about 500 ppm fluoride would be appropriate in early life, (b) whether use of fluoride-containing toothpastes should be delayed until 2 years of age, and (c) whether the concentration of fluoride in drinking water should be reduced from 1 ppm to 0.7 ppm. This debate is continuing with each country deciding its own policy, aware of the need to balance benefits of fluoridated water and fluoride toothpaste against risk of dental fluorosis. Dental fluorosis is not a disease, it is an aesthetic problem; dental caries is of medical, social and economic importance. While the age of risk of dental fluorosis is the first four years of life, benefits from water fluoridation are life-long. The first country to lower the optimal fluoride concentration in drinking water was Hong Kong when they recorded an increase in dental fluorosis. Hong Kong subsequently increased the fluoride concentration level (118, 119). The USA and Ireland have lowered the optimal concentration from 1 ppm to 0.7 ppm, and Ireland has gone further by recommending that fluoride-containing toothpastes are not used until the age of 2 years.

Australia made policy recommendations in the early 1990s and have recorded their impact (120). Toothpastes containing 400–550 ppm fluoride were introduced and recommended for young children who were advised to use smaller amounts of paste, and licking of toothpaste and use of fluoride dietary supplements were discouraged; water fluoride concentrations were unchanged. Representative samples of children in South Australia were examined using the same caries and fluorosis criteria in 1992–3 and 2002–3. A substantial fall in the prevalence and severity of dental fluorosis was recorded in both fluoridated and non-fluoridated communities (120). These authors linked these fluorosis data to caries experience to examine risk-benefit balance (117). By 2002–3, two-thirds of children used a toothpaste containing 400–550 ppm fluoride. Prevalence and severity of fluorosis had declined, as mentioned above, but there had been no change in caries experience. Population Attributable Risk (PAR) and Population Prevented Fraction (PPF) were calculated. PPF for both use of fluoridated water before 3y and use of fluoride toothpaste before 3y were both statistically significant (34 and 29, respectively) while none of the other variables (e.g., concentration of fluoride in toothpaste, brushing frequency) was significant, leading to recommendations that water fluoride concentration should be unchanged, and low-fluoride toothpaste should be used before 3y. Water fluoridation and the patterns of fluoridated toothpaste use can have different risks and benefits for oral health. Appropriate guidelines that are based on the evaluation of the risk and benefit of each component of fluoride exposure should lead to a more beneficial outcome.

In the UK, no change in the optimal concentration of fluoride in drinking water (1.0mgF/L) has been made and the use of toothpastes containing at least 1,000 ppm fluoride recommended from infancy, since no increase in aesthetically relevant dental fluorosis has been recorded (121). Against this backdrop, has been the publication of two Cochrane reviews of water fluoridation containing extensive reviews of the relation between water fluoride concentration and dental fluorosis (14, 18). These analyses have been heavily criticised for several reasons (151). First, Cochrane (14, 18) stated that with a fluoride level of 0.7 parts per million (ppm), approximately 12% of participants had fluorosis of aesthetic concern and approximately 40% had fluorosis of any level, implying that this was the degree of risk from water fluoridation. However, “fluorosis risk” is not zero in the absence of water fluoridation (Table 4) and if the intention is to quantify the risk of fluoridating water supplies, then the risk in the absence of fluoridation needs to be subtracted from the risk when water is fluoridated – a 4% increase when moving from 0.1 ppm to 0.7 ppm for dental fluorosis of aesthetic concern (Table 4). Second, a variety of indices was used, including the DDE index which, as described above, records all defects from all causes; thus inflating the proportion affected. Third, many of the 90 countries included in the dental fluorosis analyses were from hot climates – 11 from India, 10 from Africa, 6 from China and 4 from Mexico. In all these, because of different climates, optimal fluoride concentrations in drinking water will be lower (e.g., Singapore 0.5mgF/L) than in temperate climates. Thus, the risk probabilities given in Table 4 have little relevance in countries with temperate climates. Nor will this risk information be of value to countries with hot climates since risk has been diluted by inclusion of countries with temperate climates. The appropriate way is to examine caries and dental fluorosis in your own country and quantify the risk-benefit balance, as published in Australia by Do and Spencer (117) and Spencer and Do (120) and discussed above. Assessment of dental caries was discussed in Section 3.1 and dental fluorosis in Section 5.

**Table 4:** Probability of enamel defects including dental fluorosis, for all levels of defects and for levels of aesthetic concern.

Water fluoride concentration mgF/L	All levels	Aesthetic concern
0.1	0.28	0.08
0.4	0.33	0.10
0.7	0.40	0.12
1.0	0.47	0.15

Data from the 2024 Cochrane Review (14).

## 6 Water fluoridation and the environment

Environmental cost is now an important topic and should be considered when making decisions in public health. There is no convincing scientific evidence that community water fluoridation causes environmental harm when implemented at the carefully controlled levels used for public health. Fluoride is a naturally occurring mineral, already present in soil, rocks, water, and the atmosphere. Sea water contains about 1mgF/L (122). Fluoridation of public water supplies simply raises the fluoride content to the optimal level for preventing tooth decay—usually between 0.7 and 1.0 mgF/L. In regions with water naturally fluoridated, fluoride concentrations are often much higher.

Environmental assessments in the UK and internationally suggest that water fluoridation, when conducted within regulated limits, poses little or no significant risk to the aquatic environment. The UK Environment Agency, in correspondence relating to fluoridation proposals, has stated that anticipated releases of fluoride from treated waste water would not exceed its non-statutory environmental quality standard set to protect aquatic life (123). Regulatory and scientific evaluations have consistently concluded that, at concentrations used in community water fluoridation, environmental contamination of soil, air, and water is expected to be very limited (124).

A major source of confusion stems from the conflation of CWF with industrial fluoride pollution. Industrial emissions from fertiliser plants or aluminium smelting involve far higher fluoride concentrations and different chemical behaviours, which are not relevant to the dilute and controlled dosing used in water fluoridation. CWF uses regulated compounds that are safely stored, monitored, and dosed using automated systems with robust safeguards. In the UK, these systems are overseen by the Drinking Water Inspectorate (DWI) under the Water Supply (Water Quality) Regulations 2016, which require public reporting and routine inspections (125).

The study of Duane et al. (126) examined the environmental impact of water fluoridation in Ireland where it is national policy. Impact was assessed using comparative life cycle assessment (LCA) for a 5-year-old child over one year. The authors concluded that when comparing community-level caries prevention programmes, water fluoridation had the lowest environmental impact in all 16 categories and had the lowest disability-adjusted life years impact.

Reducing dental disease itself is also an environmental priority. Dental treatment generates substantial carbon emissions due to the use of materials like plastics and amalgam, as well as energy consumption and patient travel. CWF, by preventing disease upstream, indirectly reduces this environmental burden. Public Health England's 2018 carbon modelling report found that improvements in oral health can significantly lower dentistry's overall carbon footprint (127).

The European Commission's Scientific Committee on Health and Environmental Risks (SCHER) concluded in its 2011 review that there was no new evidence of hazard from fluoride in water or from the agents used to fluoridate (128). A separate 2019 environmental assessment from Canada's CADTH confirmed these findings and noted the efficiency and safety of modern fluoridation systems (129).

In summary, fluoridation not only avoids environmental harm, it contributes positively by lowering the environmental impact of oral health care. It is a clean, efficient, and well-regulated intervention which is supported by environmental, public health, and water safety bodies worldwide.

## 7 Cost of water fluoridation; technical expertise required for implementation and maintenance

Community Water Fluoridation is a well-established public health measure that requires careful technical planning, precise implementation, and ongoing monitoring. The process involves adding fluoride to public water supplies at an optimal concentration for caries prevention while ensuring safety and compliance with health regulations.

Fluoridation is achieved by introducing specific fluoride compounds into water supplies. The most commonly used substances are sodium fluoride, sodium fluorosilicate, and hexafluorosilicic acid. These compounds are chosen based on factors such as solubility, cost-effectiveness, and ease of handling. Large water systems typically use liquid fluorosilicic acid, while smaller systems may rely on dry compounds. In some cases, where naturally occurring fluoride levels exceed recommended concentrations, water treatment processes may involve reducing fluoride levels to prevent excessive exposure (130).

Ensuring the correct fluoride concentration is critical. In the UK, the optimal range is set between 0.7 and 1.0 milligrams per litre, based on government guidelines (131) and the World Health Organization (132). Water authorities monitor fluoride levels by daily testing and automated dosing systems. Independent regulatory agencies conduct periodic reviews and audits to ensure compliance with public health standards.

Safety remains a key priority in fluoridation programmes (132). Modern fluoridation systems include precise metering pumps and fail-safe mechanisms to prevent dosing errors. Operators undergo specialist training to handle fluoride compounds safely, and alarm systems are in place to detect and rectify irregularities immediately. Another consideration is the effect of fluoridation chemicals on water supply infrastructure. While fluoride itself does not corrode pipes, certain compounds may interact with water treatment materials. To mitigate this, water systems apply pH adjustment techniques and corrosion control measures.

From an environmental perspective, fluoridation chemicals are naturally occurring minerals, and their use in water treatment is carefully managed to prevent environmental impact. Regulatory bodies ensure that fluoride levels in wastewater do not exceed safe limits. Compliance with environmental standards and water quality directives ensures that fluoridation remains both a health-promoting and environmentally responsible intervention.

## 8 Implementing water fluoridation, role of government, public health ethics

### 8.1 Implementing water fluoridation

As pointed out in an earlier section, about 25 countries around the world have active water fluoridation programmes. While it is promoted by WHO as an effective, equitable, safe and cost-effective public health measure (4, 6, 40, 41), many countries have decided against water fluoridation opting, instead, for programmes such as salt fluoridation (133). Reasons for this are non-availability of suitably extensive public water supplies that are safe for drinking, technical ability to operate water fluoridation systems, and personal choice, since a person can decide whether to purchase fluoridated or non-fluoridated salt. It can be noted that in water fluoridated communities, people are usually able to purchase fluoride-low bottled water if that is their preference. Many countries, particularly low- and middle-income countries decide that scarce funds cannot be allocated to such public health programmes. Thus, water fluoridation will only be implemented if (a) caries prevalence is sufficiently high to warrant this intervention – it is almost invariably so, (b) there are extensive public water supply networks, (c) there is technical skill to install and operate water fluoridation plants, and an electricity supply to ensure its continual function, and (d) the political will to decide on this public health measure and allocate funds for installation of equipment and continuous fluoridation and monitoring. These last two are important since, as an example, there used to be extensive and effective water fluoridation programmes in the German Democratic Republic (East Germany) (15, 134) but, with the turmoil of unification of Germany, maintenance of plants became difficult and the political will to continue water fluoridation disappeared. Recently, in the USA, as mentioned earlier, the US Health and Human Services (HHS) Secretary, Robert F Kennedy Jr ordered counties of Florida and Utah to cease water fluoridation programmes, against professional advice. A summary of recent federal actions in this regard has been issued by the US Congressional Research Service (135). While WHO might urge “member states” to consider water fluoridation, each member state has to decide whether it is suitable, affordable and politically acceptable. This is a changing and challenging landscape where, on one hand, technical ability to fluoridate becomes easier and there is heightened commitment to public health, public distrust in scientists cannot be ignored, enhanced by the advent of 'social media', as exemplified by a rise in fluoride hesitancy (136).

Political decisions will be influenced by the public perception of water fluoridation and ethical considerations which includes loss of personal choice. Professional medical and dental advice to politicians is influential. For example, in the UK, the Chief Medical Officers of the four constituent countries issued a joint statement reaffirming their endorsement of fluoridation as a safe, effective, and socially equitable public health intervention that reduces oral health inequalities (137) giving reassurance to the political decision-makers and the public.

### 8.2 Public health ethics and water fluoridation

Community Water Fluoridation is underpinned by core public health ethics principles: beneficence, equity, solidarity, and proportionality (138–140). It is a population-level intervention that seeks to reduce the burden of dental disease across entire communities, especially among those at greatest risk. Because CWF requires no action by individuals to benefit, CWF is especially effective at reaching vulnerable and underserved groups, contributing to fairness and health equity.

The principle of beneficence supports CWF because it is demonstrably effective in reducing dental decay (140, 141). Importantly, the intervention poses minimal risk, with multiple international reviews confirming that when fluoride levels are kept within recommended limits, there is no credible evidence of harm.

CWF also meets the ethical principle of proportionality: the benefits to public health clearly outweigh any minor risks or burdens. Dental fluorosis, the only side effect, if it occurs it is usually mild and is a cosmetic issue not a health concern. At the same time, the burden of untreated tooth decay, particularly in children, can be severe, leading to pain, infection, lost school days, and hospital admissions for extractions under general anaesthetic.

Some opponents have raised concerns about autonomy which is the idea that individuals cannot consent to fluoride in their drinking water. This concern is a valid one, but it must be weighed against the reality that many public health interventions function on a collective level. The ethical debate around fluoridation is therefore less about absolute autonomy and more about balancing individual preference with community benefit. As Patel, Patrick, and Dyer point out in their overview and scoping review (141, 142), much of the fluoridation ethics literature has relied on models developed for clinical medicine, which focus heavily on individual consent. These frameworks often fail to capture the broader context of public health ethics, which includes principles like solidarity, justice, and intergenerational equity.

The Australian National Health and Medical Research Council (143) noted that water fluoridation demonstrates community solidarity, as it is an action taken by the government to look after the dental health of all citizens, especially children, and that it is a generally acknowledged responsibility of governments to act in support of good health in ensuring things like clean air and safe food, and many people see fluoridation in the same way (143).

This perspective has found resonance in countries like New Zealand, where scholars have defended fluoridation not just on scientific grounds, but also as a justified, ethical act of collective responsibility (144). In the UK, professional bodies such as the Royal College of Paediatrics and Child Health (145), directors of dental public health in Scotland (146), and the Chief Medical Officers (137) have consistently reaffirmed that CWF is not coercive, but proportionate and fair. This is especially evident given the pronounced disparities in oral health between different income groups. One can argue that it is unethical not to fluoridate given that CWF is effective and safe.

CWF also satisfies ethical standards of transparency and accountability. Public consultations have accompanied new or revised schemes in the UK, and statutory frameworks require open decision-making processes. Since 2022, the Health and Care Act has shifted some decision-making powers to central government, but public engagement remains a formal part of any new implementation in the UK (147, 148).

In summary, CWF stands on firm ethical ground. It protects the vulnerable, reduces inequalities, aligns with best practice in public health ethics, and is supported by a wealth of expert consensus and public accountability.

### 8.3 Views of the public on water fluoridation

Elected politicians will be much influenced by the views of the public when deciding to implement, continue or terminate water fluoridation programmes. Polling data from several countries suggest that people are generally favourable toward fluoridation when they understand that it reduces tooth decay, especially in children, and is endorsed by major health organisations like the World Health Organization, the US Centers for Disease Control and Prevention, the National Health and Medical Research Council in Australia, and the National Health Service in the UK. Where public votes have resulted in rejection of fluoridation, they have often been influenced by well-organised campaigns of misinformation or fear.

Many people remain unaware of fluoridation or do not feel strongly about it. This dynamic often allows anti-fluoridation voices to dominate public discourse. However, robust research indicates that the majority either support fluoridation or regard it as a standard, low-profile public health measure, akin to adding calcium and folic acid to flour or iodine to salt.

Over the past 25 years, several national opinion surveys have been conducted by independent research companies in the UK, including NOP, Gallup, and MORI (149, 150). These surveys used rigorous sampling techniques to accurately reflect the views of the general population. Results have consistently shown that between two-thirds and three-quarters of respondents support the addition of fluoride to drinking water if it can help reduce tooth decay. In these surveys, supporters of fluoridation typically outnumber opponents by a margin of three or four to one. Despite the persistence of public debate and the vocal presence of organised anti-fluoridation groups, there is no reliable evidence to suggest that a majority of the public opposes water fluoridation. Support can vary depending on how the question is framed, local political circumstances, and the extent of public awareness. Nonetheless, surveys indicate that support increases markedly when the public is accurately informed.

## 9 The future

The continuing, and in some cases increasing, high prevalence and severity of dental caries worldwide indicate the urgent need for effective, economic and safe upstream measures. Water fluoridation is one of these measures. It will not be suitable for all countries but it should be carefully considered. This article provides information to aid decision-making at all levels, and to inform public health workers and dental professionals on the many aspects of water fluoridation. With increasing global urbanisation, increasing infrastructure and technical skills, the future of water fluoridation looks bright. Many countries rely on the World Health Organisation for guidance on appropriate public health measures aimed at improving oral health, and WHO has consistently urged member states to consider implementing water fluoridation programmes. At the end of the WHO global action plan in 2030 (4), we will be able to see what progress has been made.
